# Impact of obesity upon prostate cancer-associated mortality: A meta-analysis of 17 cohort studies

**DOI:** 10.3892/ol.2014.2841

**Published:** 2014-12-31

**Authors:** XIAOYI ZHANG, GUIQIN ZHOU, BO SUN, GUOHUA ZHAO, DEZHONG LIU, JIAGE SUN, CHUANHAI LIU, HUI GUO

**Affiliations:** 1 Department of Urology, The Second Artillery General Hospital People’s Liberation Army, Beijing 100088, P.R. China; 2 Center of Integrated Traditional Chinese and Western Medicine, Beijing Ditan Hospital Affiliated to Capital Medical University, Beijing 100088, P.R. China

**Keywords:** prostate cancer, obesity, prospective cohort studies, incidence, mortality, meta-analysis

## Abstract

A number of epidemiological studies have suggested that obesity is associated, albeit inconsistently, with the incidence of prostate cancer (PCa). In order to provide a quantitative assessment of this association, the present study examined the correlation between obesity and the incidence and associated mortalities of PCa in an updated meta-analysis of cohort studies. The cohort studies were identified by searching the EMBASE and MEDLINE databases on January 1, 2014. The summary relative risks (RRs) with 95% confidence intervals (CIs) were calculated using random-effects models. In total, 17 studies, which included 3,569,926 individuals overall, were selected according to predefined inclusion criteria. Based upon the results of the random-effects models, obesity was not significantly correlated with the incidence of PCa (RR, 1.00; 95% CI, 0.95–1.06). However, further analysis revealed that obesity was significantly correlated with an increased risk of aggressive PCa (RR, 1.14; 95% CI, 1.04–1.25). Furthermore, an increased risk of PCa-associated mortality was significantly associated with obesity (RR, 1.24; 95% CI, 1.15–2.33), without any heterogeneity between the studies (I^2^=0.0%; P=0.847). The present study provides preliminary evidence to demonstrate that obesity is a significant risk factor for aggressive PCa and PCa-specific mortality. The low survival rates observed among obese males with PCa may be a likely explanation for this association.

## Introduction

Prostate cancer (PCa) is a heterogeneous disease. In 2012, there were an estimated 241,740 new cases and 28,170 associated mortalities, making the disease the second most commonly diagnosed cancer and the second leading cause of cancer-associated mortality in males (1). With the recent developments in imaging technology and novel prostate biopsy protocols, the diagnosis of PCa has improved markedly. At present, radical prostatectomy is the only effective treatment for such organ-confined tumors. Despite this, 20–30% of patients experience recurrence following the complete removal of a tumor, which is typically detected by a rise in serum prostate-specific antigen levels (2,3).

Obesity, which is generally measured by the body mass index (BMI), has become a worldwide public health problem. In total, >1 billion people are estimated to be overweight (BMI, ≥25), and >300 million of these are considered obese (BMI, ≥30) (4). Accumulating evidence suggests that obesity may be associated with an increased risk of cancer at several sites in the body (5). However, the association between PCa and obesity remains controversial. Certain studies have identified positive correlations between obesity and the incidence and aggressiveness of PCa (6,7). By contrast, other studies have not demonstrated such correlation between obesity and an increased incidence of PCa or associated-mortalities (8,9). The present study therefore aimed to analyze the correlation between obesity and the incidence and associated mortalities of PCa in an updated meta-analysis of cohort studies. This updated analysis of 17 cohort studies will allow for improved risk estimates compared with the previous analysis (7).

## Materials and methods

### Search strategy

The present systematic review was conducted according to the Meta-analysis of Observational Studies in Epidemiology guidelines (10). On January 1, 2014, searches were conducted using the MEDLINE and EMBASE databases for studies that had examined obesity- and PCa-associated mortalities. The search strategy used medical subject heading terms and keywords: (Obesity [All Fields] OR BMI [All Fields] OR body mass index [All Fields] OR overweight [All Fields] OR weight[All Fields]) and (‘prostate cancer’ [MeSH Terms] OR ‘PCa’ [MeSH Terms] OR ‘prostatic neoplasm’ [MeSH Terms] OR ‘prostate neoplasm’ [MeSH Terms] OR ‘cancer of the prostate’ [MeSH Terms] OR ‘prostatic cancer’ [MeSH Terms] and (risk [All Fields]) and (incidence [All Fields]) and (mortality [All Fields]) and (epidemiological studies [All Fields]). PCa was assessed using cancer registries, medical records, death certificates, and ambulatory and inpatient claims. Reference-retrieved reviews, meta-analyses and other relevant publications were also searched for study inclusion.

### Study selection

Two investigators screened titles and abstracts according to set inclusion and exclusion criteria. The primary aim of the present study was to confirm whether a positive association between obesity and the incidence of PCa existed. The criteria for study inclusion was as follows: i) A prospective or retrospective cohort design; ii) an exposure of interest of weight or BMI at baseline and/or at the end of the follow-up; iii) an outcome of interest in PCa and/or fatal PCa; and iv) publication in English. The meta-analysis included studies that reported standardized forms of relative risk (RR), risk ratio, hazard ratio or odds ratio, those with estimates on confidence interval (CI), or those that used RRs to represent various effect estimates. Due to a decreased risk of selection bias, results from cohort studies, as opposed to case-control studies, were chosen.

The studies that were excluded from the meta-analysis were those that consisted of a case-control design. In addition to these criteria, reviews, meeting abstracts, editorials and commentaries were also excluded. Other studies that were not relevant to the meta-analysis, such as non-analytical epidemiology studies, or those that had not considered obesity and PCa as primary exposures and outcomes, were also excluded. Furthermore, studies that had failed to report these estimates, or those with only univariate estimates, were excluded. In the event that there were multiple publications from the same study population, only those that had combined independent studies, examined weight or BMI as the main interest of exposure, or presented the largest number of cases or follow-up years were included.

### Data extraction

The data were extracted independently by two authors, and then cross-checked in order to reach a consensus. The following variables were recorded: Last name of the first author, publication year, country in which the study was performed, participant characteristics, sample size, measure of association, mean length of follow-up, variables adjusted for in the analysis and the risk estimates with corresponding 95% CIs. From each study, the RR estimate that was adjusted for the greatest number of potential confounders was extracted. The quality of each study was assessed using the nine-star Newcastle-Ottawa Scale (NOS) (11). The maximum quality score was 10 points, and studies with a quality score >5 points were considered high quality.

### Statistical analysis

The RR value was used to measure the association of interest. In the event that the RRs were reported separately for subgroups by different levels of BMI, the results of the subgroups (BMI, ≥30) were combined, and a common RR was calculated for the main analysis using a fixed-effects model. The highest levels were defined as ‘obesity’, and an overall pooled RR was calculated for the main analysis using a random-effects model. In studies that included BMI values that were only divided into two open-ended categories, the RR and CI estimates were assumed for the higher BMI category.

Heterogeneity among studies was measured by the Cochrane χ*^2^* test and quantified using the I*^2^* metric. The random-effects model could have lead to additional weight and wider CIs being given to smaller studies than the fixed-effects model, and so was employed when heterogeneity was present (12). All analyses were conducted using the STATA statistical software package version 11 (Stata Corp., College Station, TX, USA) with a two-sided test. P<0.05 was considered to indicate a statistically significant difference.

## Results

### Literature search

**Figure 1:**
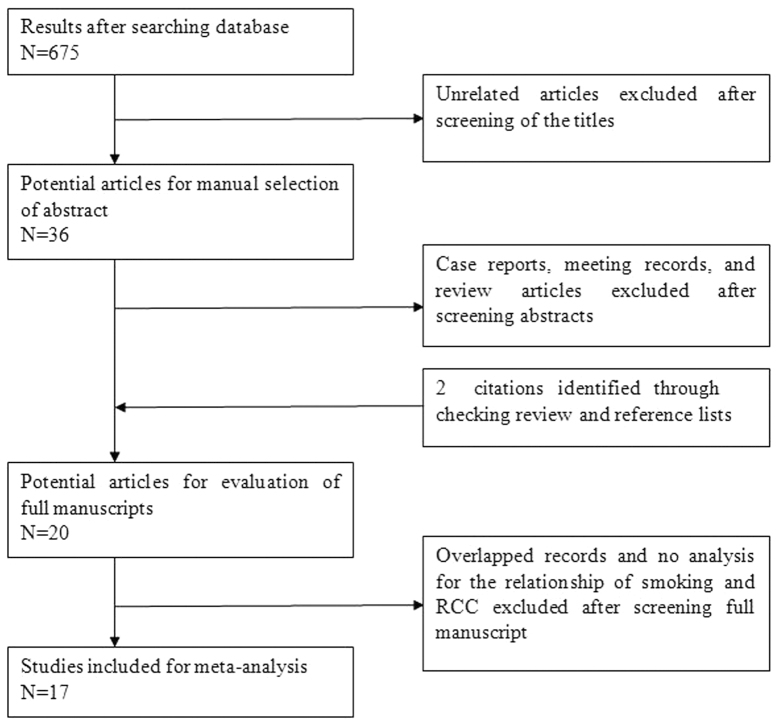
Flow diagram revealing the process of study identification, inclusion and exclusion.

The details of the literature search are presented in a flow diagram (Fig. 1). In total, 675 potentially relevant studies (378 from Medline, 265 from Embase and 32 from the Cochrane Library) were identified that had examined the association between obesity and the incidence and mortalities of PCa. Overall, 657 citations were excluded following abstract- or title-based screening. Subsequently, the 18 remaining citations, and two retrieved citations, were full-text reviewed. Following this, three studies were removed; one was a retrospective cohort study and the other two did not include RRs, a corresponding 95% CI of interest or provide sufficient data in order to calculate them. Consequently, 17 studies met all eligibility criteria and were selected for the meta-analysis.

### Study characteristics and quality assessment

The characteristics of the included studies are shown in Table I. The 17 selected studies were published between 1997 and 2012 from different populations; eight from the USA, eight from Europe and one from Oceania. The sizes of the cohorts ranged between 1,050 and 950,000 individuals. Overall, two studies were adjusted for age, and the others were controlled for certain conventional risk factors for PCa, including age and ethnicity. Each study used one of two end-points, either the incidence of PCa or PCa-associated mortality. Of the 17 cohort studies, 14 employed incidence rates, and 10 used mortality rates as the measurement of RR. The median follow-up time for the study participants ranged between 2 and 49 years. In the present meta-analysis, the median NOS score of the included studies was seven, with a range between five and nine. Furthermore, 70.59% of the studies were identified as relatively high-quality.

### Overall analyses

**Figure 2:**
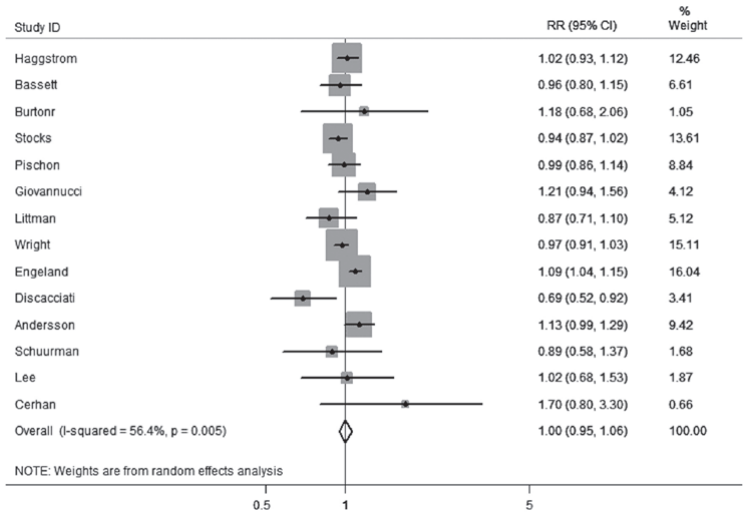
Forest plots revealing the relative risks (RR) of obesity and prostate cancer incidence. CI, confidence interval.

**Figure 3:**
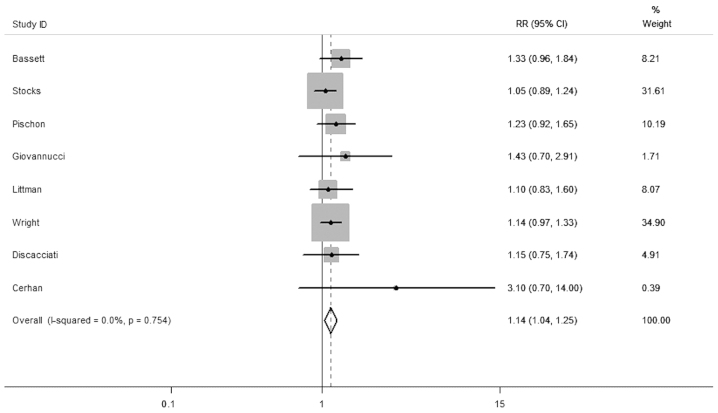
Forest plots revealing the relative risks (RR) of obesity and prostate cancer-specific mortality. CI, confidence interval.

**Figure 4:**
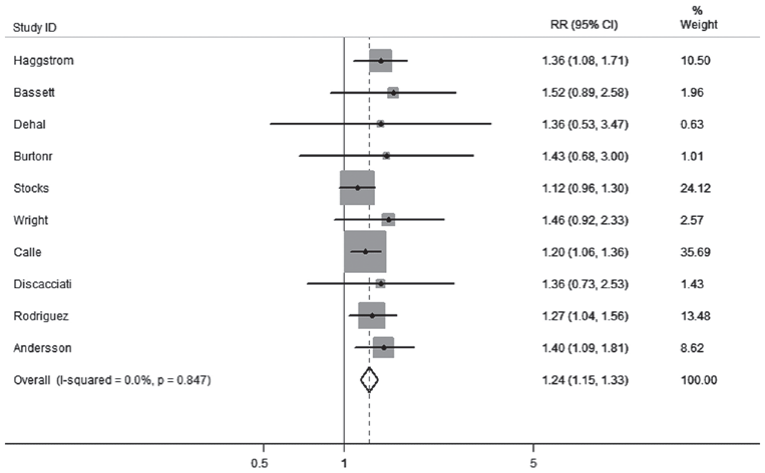
Forest plots revealing the relative risks (RR) of obesity and aggressive prostate cancer incidence. CI, confidence interval.

In total, 14 cohort studies were identified that had recorded results on obesity and the incidence of PCa. As shown in Fig. 2, obesity was not significantly correlated with PCa incidence (RR, 1.00; 95% CI, 0.95–1.06) among these studies. However, there was evidence of heterogeneity between the studies (I^2^=56.4%; P=0.005). In the subgroup analysis of aggressive disease (Gleason score between seven and 10), obesity was significantly correlated with an increased risk of aggressive PCa (RR, 1.14; 95% CI, 1.04–1.25), without any heterogeneity between studies (I^2^=0.0%; P=0.754) (Fig. 3). Overall, 11 cohort studies were identified that had presented results on obesity and PCa-associated mortalities. As shown in Fig. 4, the pooled estimates revealed a significantly higher risk of mortality from PCa when associated with obesity (RR, 1.24; 95% CI, 1.15–2.33), without any heterogeneity between studies (I^2^=0.0%; P=0.847).

## Discussion

Obesity is an increasing worldwide health concern, and an independent risk factor for various types of cancer, including colon, liver, pancreas, breast and kidney cancer (23,29). Obesity may contribute to a favorable carcinogenetic, endocrine and biochemical microenvironment for tumors (30). Despite a number of population-based observational epidemiological studies, which have examined the correlation between obesity and PCa, the association has remained controversial. A previous meta-analysis of 16 studies that was conducted in 2006 reported a positive association between obesity and the incidence of PCa (31). However, the results of the individual studies that were included in this meta-analysis differed significantly, with one demonstrating no association between obesity and PCa (32), two identifying obesity as a risk factor (22,25) and others reporting obesity as a protective factor for PCa (21,33–35). Since this meta-analysis was published, a number of complementary studies have yielded inconsistent results on the association. Given that obesity and PCa affect a large proportion of the male population, an improved understanding of the association between the two should have significant implications upon public health and clinical outcomes. The effect of obesity may not only be linked to carcinogenesis, but also to disease progression. Therefore, the present systematic review was conducted.

The present meta-analysis, which included 14 cohort studies that had examined obesity and/or the incidence of PCa, concluded that the epidemiological evidence was insufficient to confirm an association. Although obesity was not significantly correlated with the incidence of PCa (RR, 1.00; 95% CI, 0.95–1.06) among these studies, there was evidence of heterogeneity (I^2^=56.4%; P=0.005). However, following the analyses of several parameters of PCa aggressiveness and progression, obesity was revealed to be significantly associated with high Gleason scoring or clinically advanced cases of PCa. Obesity was significantly correlated with an increased risk of aggressive PCa (RR, 1.14; 95% CI, 1.04–1.25), without any heterogeneity between studies (I^2^=0.0%; P=0.754). The association between obesity and aggressive PCa was supported, as obesity was biologically linked with advanced disease. At present, the following three proposed mechanisms exist which aim to explain the association between obesity and aggressive PCa: The insulin/insulin-like growth factor-1 axis, the action of sex hormones and adipokine signaling (36). A previous meta-analysis, which included multiple prospective studies, demonstrated that although obesity exhibits a null or marginal protective effect upon localized disease, it is in fact associated with an increased incidence of advanced-stage PCa (9). The results of the present study, which suggested that obesity is associated with an increased risk of fatal PCa, are in accordance with those of the majority of prior prospective studies, which also demonstrated a statistically significant positive association between obesity and the risk of aggressive PCa.

A previous meta-analysis of prospective cohort studies conducted by Cao and Ma (7) evaluated the association between obesity and PCa-specific mortalities. The study concluded that there was a positive dose-response association between obesity and fatal PCa. The presence of a high BMI in a cancer-free population was significantly correlated with a higher risk of future PCa-associated mortalities. In addition, evidence from case-control studies was similar to that from cohort studies, which further indicated a robust association between obesity and fatal PCa. The present study reviewed evidence regarding the impact of obesity upon the rate of PCa-associated mortalities and demonstrated that obesity is a factor likely to affect disease progression. In total, 11 cohort studies were identified that had presented results on obesity and PCa-associated mortalities. As shown in Fig. 4, the pooled estimates revealed that obesity was associated with a significantly higher risk of PCa mortality (RR, 1.24; 95% CI, 1.15–2.33), without any heterogeneity between studies (I^2^=0.0%; P=0.847).

The present study was subject to unavoidable limitations (37). Firstly, high heterogeneity was detected among the studies that were reviewed in the obesity versus PCa incidence correlation analysis. Heterogeneity could not be eliminated completely, and therefore the results of the combined analysis may have also been affected. Secondly, the time expansion of the selected studies was wide, and the study design, clinical characteristics of the patients and follow-up durations were varied. Finally, as aforementioned, publication bias must always be considered in systematic reviews. The primary aim and outcome of approximately half of the included studies did not address the effects of obesity upon the incidence or mortality of PCa. The present study reported the results of only the most rigorous statistical analyses performed in each study.

Based upon the results of the present meta-analysis, obesity was identified as an additional risk factor for aggressive PCa. Furthermore, obesity was associated with a risk of PCa-specific mortality. These results, which link obesity and PCa, highlight the importance of considering BMI when screening, treating and monitoring cases of PCa. Further investigations are required in order to evaluate the effects of weight loss in patients with PCa.

## Figures and Tables

**Table I:** Study characteristics.

First author (ref.)	Year	Country	Cohort size, n	Age range, years	Follow-up, years	Obese, %	PCa cases, n	PCa mortalities, n	NOS score
Haggstrom *et al* (13)	2012	Norway	289866	44	24	11	6673	961	7
Bassett *et al* (14)	2012	Australia	16510	27–76	15	19	1374	140	6
Dehal *et al* (15)	2011	USA	7016	25–74	17	15.4	3127	44	5
Burton *et al* (16)	2010	UK	9549	-	49	-	211	111	6
Stocks *et al* (17)	2010	Sweden	336159	30±13^a^	22.2	5	10002	2601	9
Pischon *et al* (18)	2008	Europe	129502	25–70	8.5	60	2446	-	5
Giovannucci *et al* (19)	2007	USA	47750	40–75	16	100	3544	312	7
Littman *et al* (20)	2007	USA	34754	50–76	2	24	832	-	8
Wright *et al* (21)	2007	USA	287760	50–71	6	21	9986	173	7
Engeland *et al* (22)	2003	Norway	950000	20–74	21	-	33140	-	5
Calle *et al* (23)	2003	USA	404576	-	16	-	-	4004	8
Discacciati *et al* (9)	2011	Sweden	36959	45–79	10	9.69	2084	225	8
Rodriguez *et al* (24)	2001	USA	381638/434630	52/57^b^	13/14	5.7/8.4	-	1590/3622	6/6
Andersson *et al* (25)	1997	Sweden	135006	-	18	-	2368	708	6
Schuurman *et al* (26)	2000	Netherlands	58279	55–69	6.3	-	681	-	5
Lee *et al* (27)	2001	USA	8922	College^c^	5	42	439	34	6
Cerhan *et al* (28)	1997	USA	1050	65–101	11	-	71	-	5

a Mean±SD,

b median age,

c participants were all measured during their time at college, no age range is available.

PCa, prostate cancer; NOS, Newcastle-Ottawa Scale.
